# Structural dynamics of the plant hormone receptor ETR1 in a native‐like membrane environment

**DOI:** 10.1002/1873-3468.70153

**Published:** 2025-09-01

**Authors:** Moritz Lemke, Nils Alexander Lakomek, Georg Groth

**Affiliations:** ^1^ Institute of Biochemical Plant Physiology Faculty of Mathematics and Natural Sciences, Heinrich Heine University Düsseldorf Germany; ^2^ Institute of Physical Biology (IPB), Faculty of Mathematics and Natural Sciences, Heinrich Heine University Düsseldorf Germany; ^3^ Institute of Information Processing: Structural Biochemistry (IBI‐7), Forschungszentrum Jülich Germany

**Keywords:** ethylene signaling, metal‐triggered long‐range allosteric signaling, Nanodisc reconstitution, plant histidine kinase, plant hormone receptor, solution‐state NMR

## Abstract

Ethylene (C_2_H_4_) regulates plant processes, such as germination, fruit ripening, and stress responses, impacting nutrition and food quality. The membrane‐bound receptor ETR1 from *Arabidopsis thaliana* is a model for ethylene signaling, but both full‐length and the soluble cytoplasmic domain have resisted crystallization. We present high‐resolution NMR spectra of full‐length ETR1 reconstituted in lipid nanodiscs, overcoming limitations and enhancing sample uniformity. ETR1 shows high internal dynamics with regions decoupled from the transmembrane domain, possibly explaining past crystallization failures and reflecting functional flexibility. Introduction of Cu(I), an essential cofactor for ethylene binding, stiffened receptor dynamics, suggesting a stabilizing role in signal transmission. This work demonstrates nanodisc‐based strategies as powerful tools for resolving membrane protein structures in plant signaling.

## Graphical abstract

The present study unveils the structural and signaling dynamics of ETR1, a key plant ethylene receptor. Using an optimized nanodisc system and solution NMR, we captured full‐length ETR1 in a native‐like membrane environment. Our findings reveal dynamic domain uncoupling and Cu(I)‐induced rigidification, providing the first evidence of metal‐triggered long‐range allosteric signaling in a plant hormone receptor.

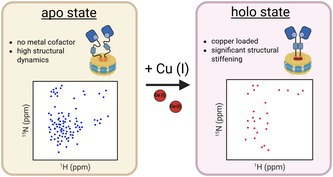

## Abbreviations

**ATP**, adenosine triphosphate

**BCA**, bicinchoninic acid

**CV**, column volume

**Cu(I)**, copper(I) ion

**DMPC**, 1,2‐dimyristoyl‐*sn*‐glycero‐3‐phosphocholine

**ER**, endoplasmic reticulum

**ETR1**, ethylene receptor 1

**Fos14**, n‐tetradecylphosphocholine (Fos‐Choline‐14)

**GAF**, cGMP‐specific phosphodiesterases, adenylyl cyclases, and FhlA domain

**HK**, histidine kinase

**HSQC**, heteronuclear single quantum correlation

**IMAC**, immobilized metal affinity chromatography

**IPTG**, isopropyl β‐D‐1‐thiogalactopyranoside

**MSP**, membrane scaffold protein

**ND**, nanodisc

**NMR**, nuclear magnetic resonance

**OD600**, optical density at 600 nm

**PBS**, phosphate‐buffered saline

**PMSF**, phenylmethylsulfonyl fluoride

**SEC**, size exclusion chromatography

**TMD**, transmembrane domain

**TEV**, tobacco etch virus (protease)

**Tris**, tris(hydroxymethyl)aminomethane

**TROSY**, transverse relaxation optimized spectroscopy

Ethylene (C_2_H_4_), a small gaseous hydrocarbon, is a pivotal plant hormone implicated in numerous developmental and stress‐related processes with high agronomic and economic implications [1, 2, 3]. Consequently, a comprehensive understanding of this multifaceted plant hormone is imperative for optimizing the cultivation, storage, and transport of fruits and vegetables, thereby extending their shelf life and minimizing postharvest losses. The ethylene response in plants is mediated by membrane‐bound receptors, with ethylene receptor 1 (ETR1) from *Arabidopsis thaliana* being the most studied [4, 5]. ETR1 and further members of the *Arabidopsis* ethylene receptor family (ETR2, EIN4, ERS1, and ERS2) are multidomain proteins that share significant homology with prokaryotic two‐component signaling systems [2, 4]. Among these, ETR1 (738 residues, 82.5 kDa per monomer) forms a homodimer. It comprises four functional domains per monomer: an N‐terminal transmembrane domain (TMD), followed by a GAF domain, a histidine kinase domain [consisting of a dimerization histidine phosphotransfer (DHp) and a catalytic domain (HK, which binds ATP)], and a C‐terminal receiver domain (Table S1). The TMD anchors the receptor in the endoplasmic reticulum (ER) membrane and contains the ethylene‐binding site, where the plant hormone is recognized via a Cu(I) cofactor [6, 7, 8]. Unlike many membrane receptors, ETR1 does not possess a soluble signal‐binding domain. Instead, ethylene binding occurs via the TMD embedded in the lipid membrane. Although individual soluble domains of ETR1 or related receptors have been crystallized [9], including the receiver domain (PDB code: 4PL9), the catalytic domain (PDB code 1DCF), and the histidine kinase domain of related ERS1 (PDB code 4MT8), neither full‐length ETR1 (including the N‐terminal TMD) nor the full cytoplasmic soluble part (without the TMD) has been crystallized, despite decades of effort. The TMD is thought to serve as the primary dimerization interface [10]. However, additional domains, including the GAF domain and histidine kinase domain, have also been implicated in receptor dimerization [9, 11].

The GAF domain, located adjacent to the N‐terminal transmembrane region, likely stabilizes the receptor in the functional dimeric state and bridges interactions within the ethylene signaling network [12]. The following histidine kinase domain, a hallmark of two‐component systems, mediates receptor autophosphorylation and downstream signaling. The final receiver domain at the C terminus may not be directly involved in signal output, but rather serves a regulatory role in downstream signaling. As outlined above, the structure of the intact full‐length ETR1 embedded in a lipid bilayer environment, and the interplay between the individual receptor modules, remains unknown, yet is imperative for elucidating the mechanisms of signal recognition and signal transfer within the receptor and for downstream signaling.

Indeed, the study of membrane proteins is fraught with challenges due to the intrinsic hydrophobic character of these proteins. They require a lipid environment to maintain their native conformation and function, making them difficult to handle and study in aqueous solutions [13]. One of the major obstacles in studying ETR1 has been its exceptional resistance to solubilization from the bacterial membrane following heterologous expression. Experimental findings demonstrated that ETR1 can only be efficiently extracted using a very limited subset of detergents, specifically *n*‐Tetradecyl‐phosphocholine (Fos‐Choline‐14) and *n*‐Hexadecyl‐phosphocholine (Fos‐Choline‐16). However, prolonged exposure to these detergents can result in unfavorable effects, such as the destabilization of proteins over time or interference with downstream structural and functional analyses [14]. These factors have contributed to the difficulty of obtaining high‐resolution structural insights into the intact receptor.

Recently, we successfully incorporated the full‐length ETR1 receptor into synthetic membrane systems known as lipid nanodiscs (NDs). These nanosized membrane mimetics provide a more natural lipid environment for the receptor, allowing for the overcoming of some of the limitations posed by traditional detergent‐based solubilization methods [15, 16, 17, 18, 19]. Using biophysical techniques such as Small‐Angle X‐ray Scattering (SAXS) and nuclear magnetic resonance (NMR) spectroscopy, we were able to characterize the receptor in a native‐like membrane environment, eliminating the destabilizing effects associated with detergents [20]. However, a major limitation of our previous study was the inability to efficiently separate ETR1‐loaded nanodiscs from receptor‐free NDs. This problem was likely related to the steric hindrance and shielding of the embedded receptor's 10×‐Histidine tag by the surrounding lipid molecules of the NDs, preventing effective binding to the nickel‐NTA resin. Consequently, a substantial proportion of empty NDs emerged, impeding the acquisition of precise data due to a reduction in the signal‐to‐noise ratio.

## Materials and methods

### Expression of ethylene receptor ETR1

Full‐length *Arabidopsis thaliana* ETR1 was heterologously expressed in *Escherichia coli* C41(DE3) ∆(ompF‐acrAB) [21] using a pETEV16b vector. Cultures were grown in 2YT medium with 100 μg·mL^−1^ ampicillin at 37 °C and induced with 0.5 mm IPTG at an OD600 of 0.5, followed by overnight expression at 16 °C. Cells were harvested 18 h after induction by centrifugation (7500× ***g***, 15 min, 4 °C), shock‐frozen in liquid N_2,_ and stored at −20 °C. For NMR analyses, M9 mineral medium (33.7 mm Na_2_HPO_4_, 22.0 mm KH_2_PO_4_, 8.55 mm NaCl, 0.4% glucose monohydrate, 1 mm MgSO_4_, 0.3 mm CaCl_2_, 1 μg·L^−1^ biotin, and 1 μg·L^−1^ thiamine) was complemented with 100× trace element solution (13.4 mm EDTA, 3.1 mm FeCl_3_, 0.62 mm ZnCl_2_, 76 μm CuCl_2_, 42 μm CoCl_2_, 162 μm H_3_BO_4_, and 8.1 μm MnCl_2_), 1000× MEM vitamin solution (#11120052; Thermo Fischer, Waltham, MA, USA), 100 μg·mL^−1^ ampicillin, and 1 mg·mL^−1^ 15NH_4_Cl and used for the main culture.

### Membrane preparation and solubilization

Frozen cell pellets were thawed and resuspended in lysis buffer at a ratio of 5 mL per gram of wet cell pellet. The lysis buffer consisted of 1× PBS (pH 8.0; prepared from a 10× stock containing 140 mm NaCl, 2.7 mm KCl, 10 mm Na_2_HPO_4_, and 1.8 mm KH_2_PO_4_), supplemented with 10% (w/v) glycerol and 0.002% (v/v) PMSF. All buffers used throughout the membrane preparation procedure contained 0.002% (v/v) PMSF to inhibit proteolytic degradation. Cell disruption was carried out using a high‐pressure cell disruptor (2.4 kbar). The lysate was collected in the presence of DNase I. After removal of cell debris and inclusion bodies (14 000× ***g***, 30 min, 4 °C), membranes were pelleted by centrifugation (40 000× ***g***, 30 min, 4 °C), washed, flash‐frozen in liquid nitrogen, and stored at −80 °C. Membrane pellets were thawed rapidly at room temperature and immediately resuspended in solubilization buffer consisting of 50 mm Tris/HCl, pH 7.8, 200 mm NaCl, 1.5% (w/v) *n*‐tetradecylphosphocholine (Fos14), and 0.002% (w/v) PMSF at 4 °C for 1 h. Insoluble material was removed by ultracentrifugation (100 000× ***g***, 65 min, 4 °C).

### Purification of ETR1

The solubilized ETR1 was purified by immobilized metal affinity chromatography (IMAC) using a 5 mL HisTrap™ HP column pre‐equilibrated with Fos14‐containing buffer (0.03%). Following sample loading and washing, chaperone contaminants were removed via an ATP wash step (10 mm ATP, 50 mm KCl, and 20 mm MgCl_2_). ETR1 was eluted in a stepwise gradient up to 250 mm imidazole. All chromatography steps were conducted at 4 °C. Eluted fractions were concentrated (50 kDa MWCO), desalted on PD‐10 columns, and analyzed by SDS/PAGE. Protein concentration was determined by UV absorbance at 280 nm using a NanoQuant plate (Tecan Group Ltd., Männedorf, Switzerland) in combination with an Infinite® multimode microplate reader (Tecan Group Ltd.). Measurements were performed in triplicate, and concentrations were calculated using the theoretical extinction coefficient of the ETR1_10×His construct (ε = 58 495 M^−1^ cm^−1^).

### Copper loading of ETR1

Purified ETR1 (96.8 μm) was incubated stepwise with copper‐loading buffer (50 mm Tris–HCl pH 7.8, 200 mm NaCl, 2.5 mm bicinchoninic acid (BCA), 1 mm CuCl, 20 mm ascorbate). Aliquots of copper‐loading buffer were added sequentially until no further spontaneous decolorization of the solution was observed and a stable violet color, characteristic of the BCA–Cu(I) complex, had developed. Excess unbound Cu–BCA was removed via desalting (PD‐10), and the protein was exchanged into storage buffer (50 mm Tris–HCl, 200 mm NaCl, 0.03% Fos14, and 0.002% PMSF).

### Expression, purification and digestion (TEV) of MSP1E3D1

MSP1E3D1 was expressed according to [22] in *E. coli* BL21(DE3) carrying pET16_MSP1E3D1 in TB medium. Expression was induced with 1 mm IPTG at an OD600 of 0.8, followed by 3 h incubation at 37 °C. Cells were harvested by centrifugation (7000× ***g***, 15 min, 4 °C). Afterward, the cell pellet was resuspended in MSP lysis buffer (20 mm sodium phosphate, pH 7.4, 100 μg·mL^−1^ lysozyme, 1% Triton X‐100, 1 Roche protease inhibitor tablet (Roche, Basel, Switzerland) per 50 mL, DNase I, and antifoam). Cells were lysed using a high‐pressure cell disruptor (Constant Systems, Daventry, UK) at 2.4 kbar. The lysate was clarified by centrifugation (40 000× ***g***, 45 min, 4 °C), and the supernatant was subjected to IMAC on HisTrap HP columns (Cytiva, Marlborough, MA, USA) pre‐equilibrated with lysis buffer. MSP1E3D1 was eluted from these columns with 500 mm imidazole. The pooled eluate was dialyzed three times against cleavage buffer (50 mm Tris–HCl, pH 8.0, 0.5 mm EDTA, and 1 mm DTT), with dialysis steps performed sequentially for 1 h at room temperature, 1 h at 4 °C, and then overnight at 4 °C. Following buffer exchange, protein concentration was determined by UV absorbance at 280 nm. TEV protease was added at a molar ratio of 1 : 100 (TEV : MSP1E3D1), and the cleavage reaction was incubated overnight at 4 °C. Postdigestion, the mixture was applied to a HisTrap HP column to remove His‐tagged species, including uncleaved protein, TEV protease, and free His‐tag. The cleaved MSP1E3D1 was collected in the flow‐through, concentrated using a 10 kDa MWCO centrifugal concentrator (Merck Millipore, Burlington, MA, USA), and further purified by size exclusion chromatography using a Superdex 200 Increase 10/300 GL column (Cytiva, Marlborough, MA, USA) equilibrated with 20 mm Tris–HCl, pH 7.4, 100 mm NaCl, and 1 mm DTT. Protein purity was assessed by SDS/PAGE. The final concentration of MSP1E3D1 was quantified by UV spectrophotometry at 280 nm using a theoretical extinction coefficient of ε₍₂₈₀₎ = 29 840 M^−1^ cm^−1^.

### Preparation of Nanodiscs

DMPC (1,2‐dimyristoyl‐*sn*‐glycero‐3‐phosphocholine, Avanti Lipids #850345) lipid films were prepared by chloroform evaporation under reduced pressure in a rotary vacuum evaporator (40 °C). To remove the last traces of chloroform, a vacuum of 50 mbar was applied for at least 20 min. The DMPC lipids were then dissolved with assembly buffer (50 mm Tris–HCl pH 8.0, 200 mm NaCl, and 0.45% Fos‐Choline 14) using a vortexer and an ultrasonic bath and adjusted to a working concentration of 6.5 mm. Ni‐INDIGO beads were used to immobilize ETR1. Shock‐frozen ETR1 was thawed and incubated with Ni‐beads for 20 min at room temperature in a rotary mixer. Binding was visually confirmed by the color shift of the beads from blue to yellow‐green. To initiate nanodisc assembly, MSP1E3D1 and DMPC were added to the ETR1–Ni‐bead complexes at final molar ratios of 2 : 8 : 1140 of ETR1, MSP1E3D1, and DMPC, respectively. The Fos14 concentration was adjusted to 0.45% (w/v), and the mixture was incubated at 28 °C for 1 h in a rotary mixer. Bio‐Beads SM‐2 (Bio‐Rad, Hercules, CA, USA) were then added in several portions (1 g·mL^−1^ wet beads) over a period of 7–8 h to remove detergent, followed by overnight incubation at 28 °C.

### Purification of ETR1‐Nanodiscs

After the overnight assembly reaction, bead mixture was transferred to gravity flow columns packed with the Ni‐INDIGO beads and washed with 10 CV each of wash buffers I (50 mm Tris–HCl pH 8.0, 200 mm NaCl) and II (same with 20 mm imidazole). ETR1‐nanodiscs were eluted with elution buffer (50 mm Tris–HCl, 200 mm NaCl, and 500 mm imidazole) in 0.2–0.5 CV steps. Protein concentration in elution fractions was monitored by Bradford assay, and peak fractions were pooled, concentrated (MWCO 100 kDa), and stored at 4 °C.

### Size exclusion chromatography

Purified ETR1‐nanodiscs were subjected to SEC using a Superose™ 6 Increase 10/300 GL column equilibrated in SEC buffer (50 mm Tris–HCl pH 8.0, 200 mm NaCl). To determine their molecular weight, the column was calibrated with molecular weight standards dextran blue, thyroglobulin, ferritin, conalbumin, ovalbumin, ribonuclease A (#28403842, #28403841; Cytiva) according to the manufacturer's instructions (Fig. S3).

### NMR spectroscopy

Two‐dimensional 1H–15N TROSY‐HSQC (Transverse Relaxation Optimized Spectroscopy Heteronuclear Single Quantum Correlation) spectra of ^15^N ETR1 reconstituted in DMPC lipid nanodiscs were recorded at 10 °C using a Bruker 900 MHz AVANCE neo spectrometer equipped with a 1H/15N/13C cryoprobe. Spectra were acquired using 1024 complex points in the 1H dimension and 75 in the 15N dimension, with a total acquisition time of ~16 h per spectrum. Spectral widths were 16.34 ppm (1H) and 35 ppm (15 N), and carrier frequencies were centered at 4.7 ppm (1H) and 117 ppm (15 N). Each increment was recorded with 384 scans and a 1 s recovery delay. Experiments were conducted back‐to‐back for Cu(I)‐bound and apo forms of ETR1.

NMR data were processed in Bruker TopSpin 4.4 with cosine‐squared window functions and zero‐filling to 2048 × 1024 points. Spectra were visualized and analyzed using ccpn analysisassign 3.0.4.

## Results

**Fig. 1:**
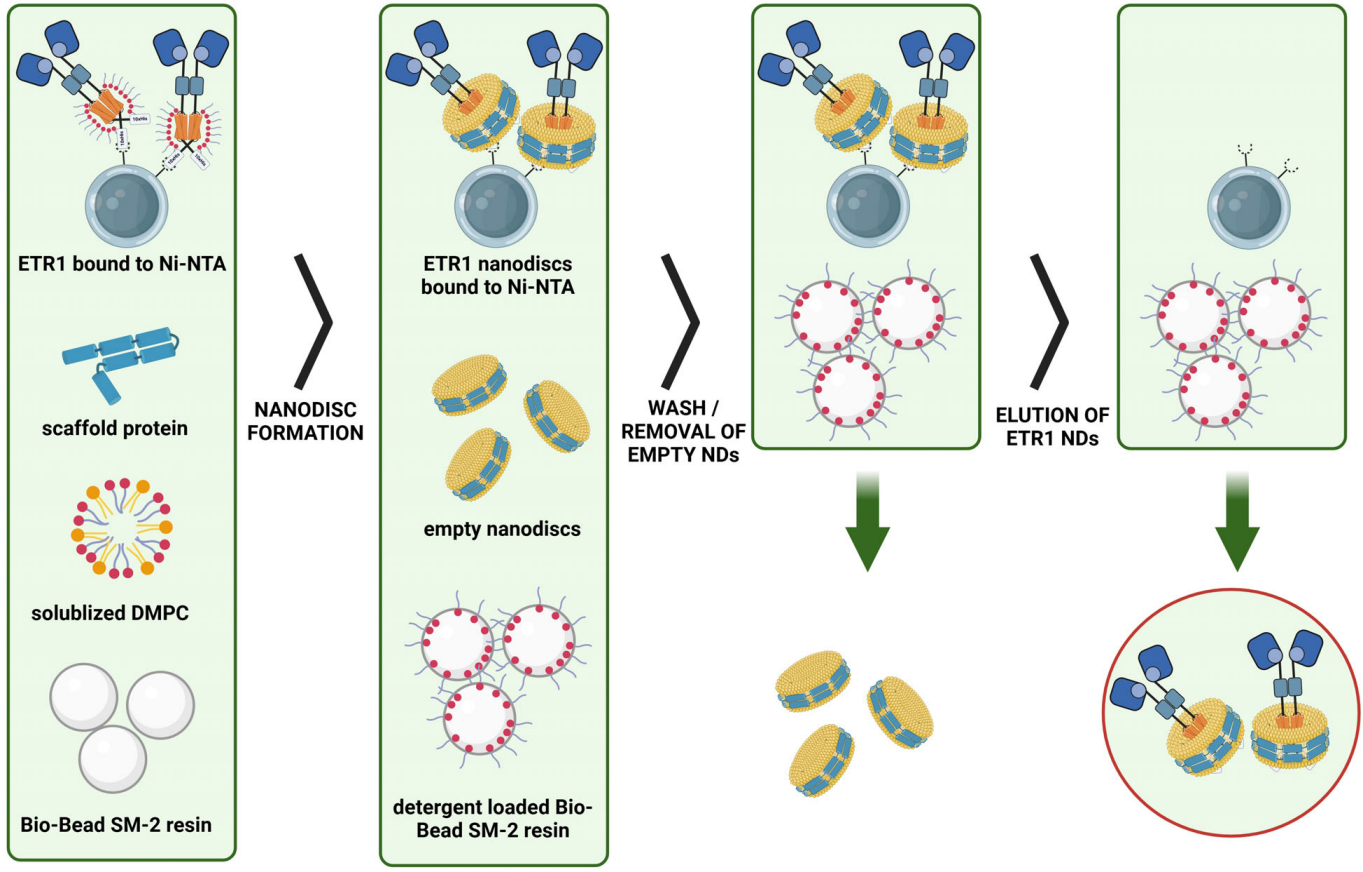
Schematic overview of the on‐column assembly process. Initially, ethylene receptor 1 (ETR1), bound to Ni‐agarose beads, is incubated with scaffold protein, solubilized 1,2‐dimyristoyl‐*sn*‐glycero‐3‐phosphocholine (DMPC) lipids, and Bio‐Beads in the assembly mixture. As the Bio‐Beads gradually absorb the detergent, both ETR1‐loaded and empty nanodiscs (NDs) are formed. Subsequently, unbound empty NDs are removed, and ETR1‐loaded NDs are eluted from the column material.

To enable more comprehensive and advanced NMR analyses of the receptor, we developed an optimized on‐column assembly protocol for lipid nanodiscs. In this method, ETR1 fitted with an N‐terminal 10×His‐Tag was expressed in *E. coli* C41ΔΔ cells [21], solubilized from the bacterial membranes with the zwitterionic detergent, Fos‐Choline‐14, and purified by IMAC. Prior to nanodisc assembly, the purified receptor was bound to a Ni‐INDIGO™ agarose resin (Cube Biotech, Monheim, Germany). This strategy was implemented to prevent lipid molecules from occluding the His‐tag, a critical element for subsequent purification and analysis (see Fig. 1). Additionally, the method allows for more precise control over the stoichiometry of loaded and empty NDs, thereby optimizing the balance required for efficient assembly. Further measures were implemented to reduce the risk of receptor aggregation, particularly during detergent removal, which is a critical phase for maintaining NDs stability and described in the following. This streamlined protocol has been shown to enhance the efficiency of ND formation and ensure the structural integrity of the incorporated proteins, making it a valuable approach for downstream biochemical and biophysical analyses of membrane proteins.

Typically, sodium cholate is employed to solubilize phospholipids for ND assembly [22, 23]. In the current study, we use Fos‐Choline‐14 as the sole detergent for lipid solubilization (DMPC lipids) and the target protein ETR1 to avoid a significant difference in critical micelle concentrations (cmc) between sodium cholate (0.54% w/v) and Fos‐Choline‐14 (0.0046% w/v). This point proved critical to circumvent a two‐step assembly process, in which empty NDs would initially form upon dropping below sodium cholate's cmc, making the subsequent migration of ETR1 from Fos‐Choline micelles into these preformed NDs necessary. Using Fos‐Choline‐14 exclusively ensured that ETR1 remained encapsulated in its micelles until the detergent concentration decreased below its own cmc. Consequently, ETR1 integration and ND assembly occurred simultaneously. Then, ETR1 assembles directly into NDs, effectively circumventing the formation of intermediate empty NDs. Furthermore, a substantial enhancement in yield and efficiency of ND reconstitution was observed when the incubation temperature was raised to 28 °C, a temperature notably higher than the typical room temperature applied in standard assembly protocols [24] and well above the lipid phase transition temperature of DMPC (Tm = 24 °C). Prior to the assembly process, the receptor was bound to Ni‐INDIGO column material, thereby facilitating the effective removal of excess ND components, empty NDs, and precipitate.

**Fig. 2:**
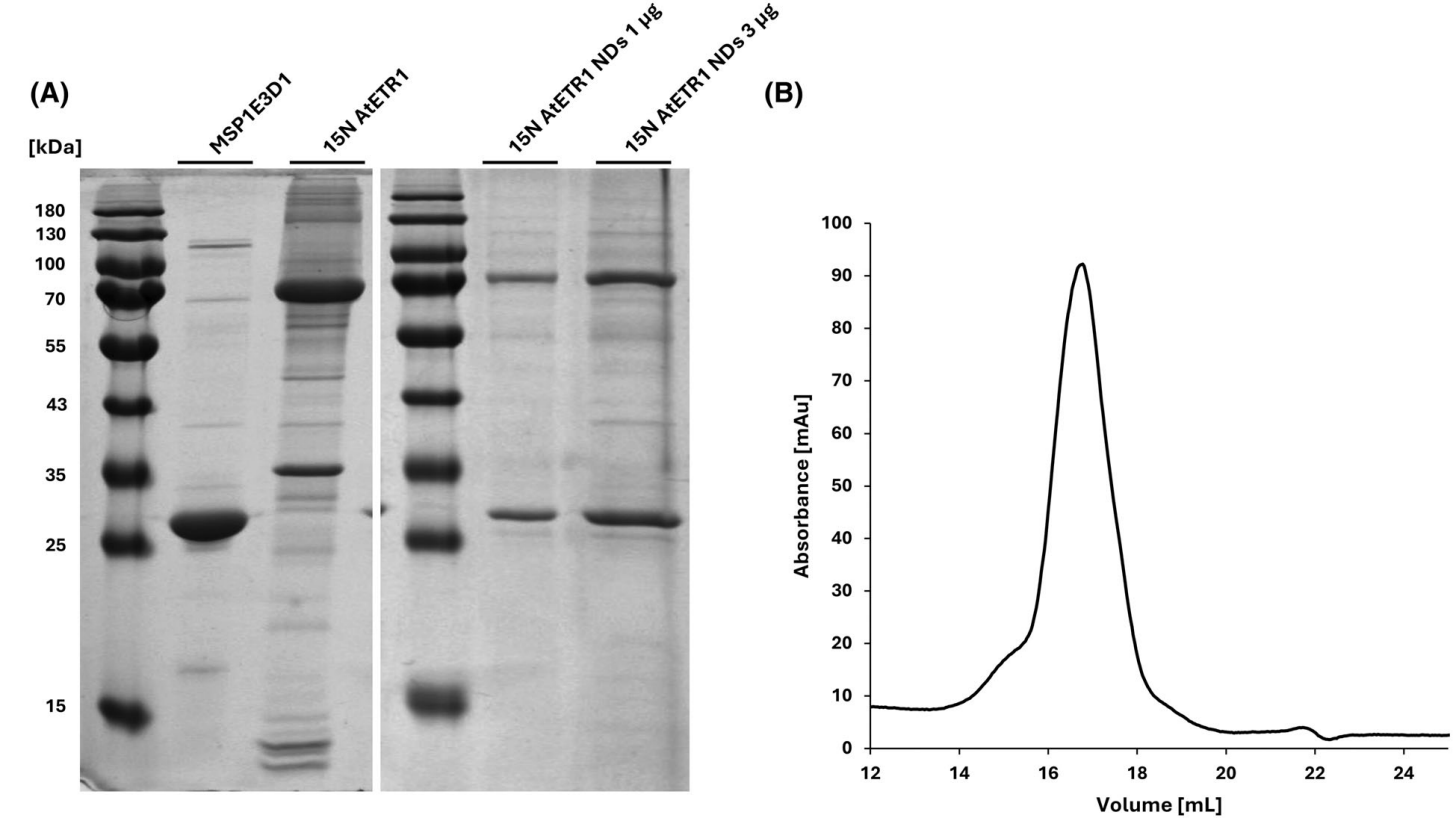
(A, left) Representative SDS/PAGE of ethylene receptor 1 (ETR1) nanodiscs (NDs), and (A, right) N15 isotope labeled ETR1 NDs. The experiment was performed in triplicate under identical conditions. (B) Size exclusion chromatography of ETR1 NDs using a Superose® 6 Increase 10/300 GL column at a flow rate of 0.3 mL·min^−1^. Based on calibration with protein standards (thyroglobulin, ferritin, conalbumin, ovalbumin, and ribonuclease A) the elution volume of ETR1‐NDs corresponds to a Mw of ~450 kDa. The shown chromatogram is representative of two independent SEC runs.

To assess the efficiency of ND reconstitution and the structural integrity of ETR1, we first analyzed the purified samples using SDS/PAGE and size exclusion chromatography. The SDS/PAGE analysis revealed nearly equivalent band intensities (1 : 1.16) for MSP1E3D1 and ETR1 (see Fig. 2A), suggesting successful separation of empty NDs from ETR1‐NDs. Subsequent size exclusion chromatography confirmed sample homogeneity, showing a near‐monodisperse peak (Fig. 2B) and an elution volume of 16.77 mL corresponding to ~450 kDa, matching the estimated weight of a nanodisc containing two ETR1 molecules. This supports the conclusion that ETR1 remains dimeric within the nanodisc environment.

**Fig. 3:**
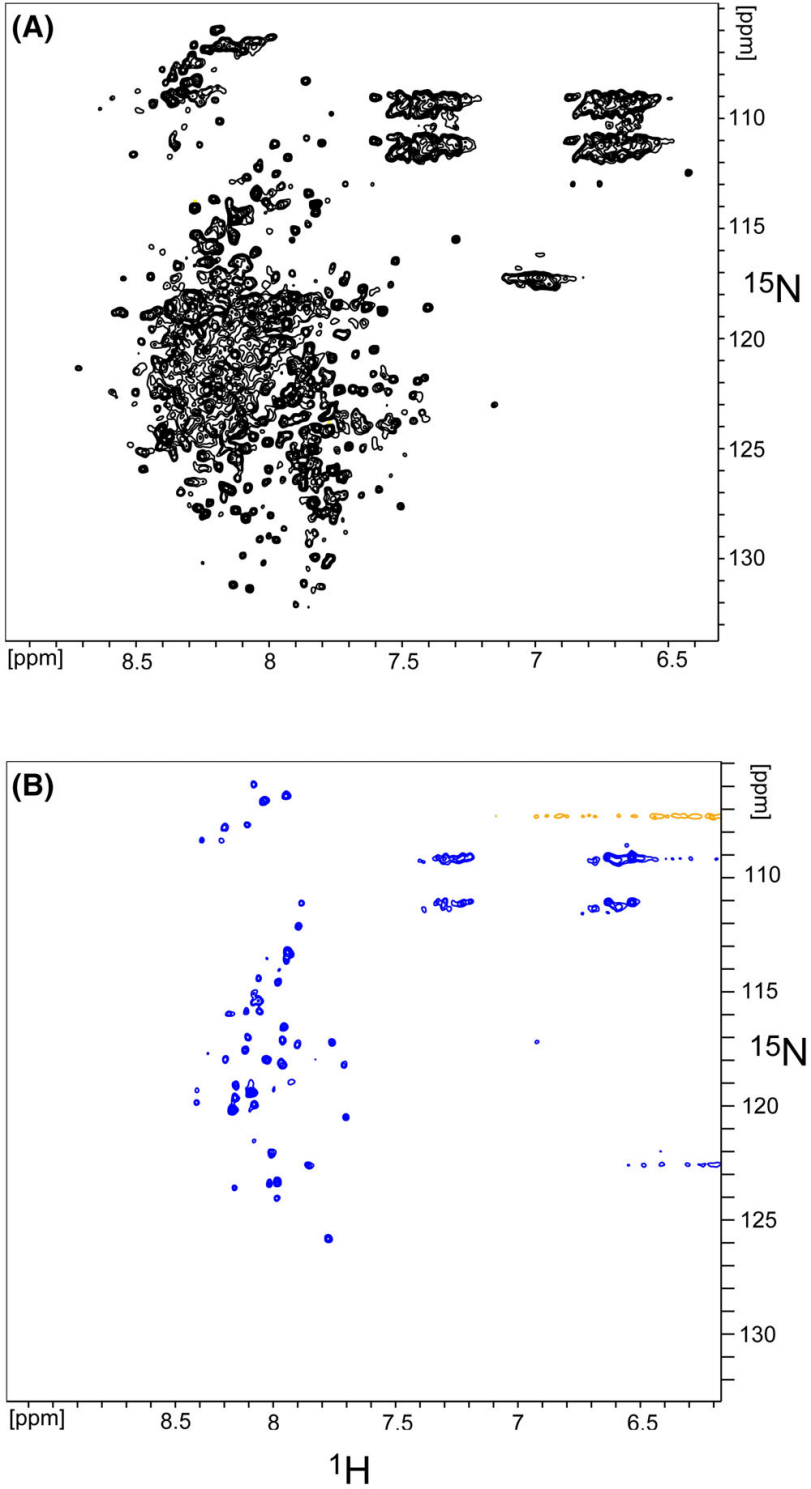
[^1^H, ^15^N] Transverse relaxation optimized spectroscopy (TROSY)—Heteronuclear Single Quantum Correlation (HSQC) spectrum of ^15^N ethylene receptor 1 (ETR1) embedded in 1,2‐dimyristoyl‐*sn*‐glycero‐3‐phosphocholine (DMPC) lipid nanodiscs: (A) in the absence and (B) in the presence of Cu(I). Those two‐dimensional [^1^H, ^15^N] spectra can be considered *fingerprint* spectra of the protein, representing the conformational and dynamic state of the protein. Spectra were recorded back‐to‐back with NS = 384 scans. (Reproducible spectra were recorded with fewer scans and a lower signal‐to‐noise ratio. The signal pattern in the region around ^1^H 7 ppm and ^15^N 110 ppm originates from side‐chain resonances and does not belong to the backbone).

To gain deeper insights into the structural state and dynamics of the ethylene receptor in the ND native‐like membrane environment, the receptor was isotopically labeled with nitrogen‐15 (N15), and two‐dimensional ^1^H–^15^N NMR correlation spectra were acquired. Figure 3A shows the two‐dimensional ^1^H,^15^N TROSY‐HSQC spectrum of homodimeric ^15^N ETR1 reconstituted in DMPC lipid ND, recorded at 900 MHz and 10 °C.

The ETR1 monomer comprises 738 residues. In the absence of transverse relaxation, a theoretical maximum of 706 backbone [^1^H,^15^N] resonances could be expected (738 minus 31 prolines and the N terminus, which cannot be detected). However, given the high molecular weight of about 450 kDa of the ETR1 homodimer embedded in lipid nanodiscs, estimated by size exclusion chromatography combined with multi‐angle light scattering [20], it is anticipated that only a limited number of visible NMR resonances (belonging to the most dynamic residues) will be observed in the solution‐state NMR spectra, due to the so‐called overall tumbling limitation of solution‐state NMR. The higher the molecular weight of a (bio)molecule, the slower the rotational diffusion (overall tumbling). This, in turn, leads to increased transverse relaxation and, as a direct consequence, to the broadening of the NMR resonances. As a rule of thumb, for a molecular weight above 200 kDa, the NMR resonances of the protein backbone become broadened below the detection threshold, rendering them invisible [25]. Conversely, side chains are more flexible and remain visible up to 1 MDa [26].

Contrary to our initial expectations, the spectrum shown in Fig. 3A reveals about 350 well‐resolved backbone resonances, in a chemical shift range from 6.4 to 8.7 ppm in the ^1^H dimension and 105 to 134 ppm in the ^15^N dimension, many of them located in the central overlap region. The chemical shift range indicates low visibility of the ETR1 β‐sheet region (part of the GAF domain), but would be in line with the presence of mainly α‐helical, loop, and intrinsically disordered regions. Previously recorded CD spectroscopy as well as functional assays argue for a well‐folded and mainly α‐helical ETR1 structure [27, 28]. Enhanced detectability of peaks can be attributed exclusively to the high internal structural dynamics of the ETR1 receptor protein. Such elevated internal dynamics would dynamically decouple part of the protein from the rigid body of the protein and the lipid nanodisc. Our observation indicates that at least half of the protein exhibits high internal dynamics. For this, multiple ETR1 domains must undergo dynamic decoupling from the transmembrane region and lipid ND. Indeed, several extended loop regions have been reported for the DHp and catalytic domain (CA) [9]. As the N‐terminal transmembrane region is embedded in the lipid ND and is unlikely to be highly dynamic, we conclude that the dynamics (visible in the NMR spectrum) refer to the C‐terminal part of the receptor, most likely involving the CA and receiver domains which are C‐terminal of the dimerization interface and connected by extended loop regions to the remainder of the protein. The catalytic domain (G408‐S589) and receiver domain (K611‐L729), connected by a long loop region (S590‐L610), are located C‐terminal of the DHp. Dynamic decoupling of both domains from the remainder of the N‐terminal part of the protein would be consistent with the presence of about 350 visible resonances. The [^1^H, ^15^N] backbone resonances of glycine residues are located in a very characteristic spectral region, frequently between 105 and 110 ppm in the ^15^N dimension. Table S1 shows the ETR1 sequence with glycine residues highlighted. A total of 36 glycine residues is present in the ETR1 sequence. Figure S1 provides a magnified view of the ETR1 glycine region of the [^1^H, ^15^N] spectrum shown in Fig. 3A. A total of about 20 intense glycine resonances are observed in this region. This observation is consistent with the C‐terminal catalytic domain and receiver domain being visible. It would support the hypothesis that these domains exhibit a high degree of flexibility, for example, enabled by the flexible linkers connecting these domains. Flexibility of the receiver domain was previously indicated by SAXS data [9].

All NMR measurements described so far were performed on the apo form of the receptor that is, in the absence of its physiological cofactor Cu(I). An important point in this context is the absence of Cu(I) in our purified recombinant ETR1 protein, despite its extremely tight binding affinity (Kd ~ 1.3 × 10^−15^ M). This initially counterintuitive observation is explained by the expression system: ETR1 was heterologously expressed in *E. coli*, which lacks copper chaperones such as ATX1 that are present in eukaryotes and are essential for targeted Cu(I) delivery. In eukaryotic cells, ATX1 and its homologs (e.g., CCH in plants) bind Cu(I) tightly and transfer it to specific transporters such as RAN1, which subsequently deliver the metal to ethylene receptors [29]. In contrast, *E. coli* relies on detoxification and efflux systems (e.g., CopA, CueO, and CusCFBA) to maintain extremely low cytosolic Cu(I) levels. As such, the bacterial cytoplasm is not conducive to the spontaneous incorporation of Cu(I) into metalloproteins, particularly in the absence of external copper supplementation. This interpretation aligns with earlier findings by [7], who showed that functional ethylene binding by yeast‐expressed ETR1 required the addition of 300 μm CuSO_4_. In our expression and purification protocol, no such copper supplementation was used, and the protein was purified under native conditions without any denaturation or refolding steps (see Materials and Methods section). Therefore, the absence of Cu(I) is not due to misfolding or loss of structural integrity, but rather to environmental limitations during expression.

In the presence of ETR1 cofactor Cu(I), the NMR spectra, however, exhibit a dramatic change: Fig. 3B shows the [^1^H, ^15^N] TROSY‐HSQC spectrum of a ^15^N‐labeled ETR1 nanodisc sample of the same batch in which ETR1 was preloaded with Cu(I) prior to nanodisc reconstitution to ensure defined and complete metal loading. To achieve this, detergent‐solubilized ETR1 was titrated stepwise with Cu(I)–BCA complex in 5 μL increments. Decolorization of the violet Cu(I)–BCA solution was observed up to a total volume of 45 μL, indicating effective binding of Cu(I) to the receptor. A persistent violet color reappeared only after the 50 μL addition, suggesting saturation of the high‐affinity Cu(I)‐binding sites. These results are consistent with a 1 : 1 binding stoichiometry and confirm that the receptor was in the apo state before loading. Excess Cu(I)–BCA was subsequently removed by gel filtration (see Methods for details). Importantly, these findings also demonstrate that ETR1 retains its Cu(I)‐binding capability after purification.

Copper was added to the samples to investigate its role as a cofactor required for ethylene binding, coordinating with key residues in the transmembrane region to facilitate hormone perception. Previous studies have suggested that Cu (I) binding not only enables ethylene recognition but may also influence the structural dynamics of the receptor [6, 30]. Only about 40 well‐resolved isolated resonances remained visible in the spectrum in the presence of Cu (I). The spectrum in 3B starkly contrasts with the one in 3A in the absence of Cu (I). While the spectrum in 3A points to substantial internal dynamics of at least half of the protein (belonging to the 350 visible resonances), only 40 backbone resonances remained visible in the presence of Cu (I). The disappearance of all other resonances (about 300, corresponding to 300 residues) points to a substantial loss of internal dynamics and, therefore, stiffening of the ETR1 in the presence of Cu (I). Such rigidification is further substantiated by the 1D spectrum of the first increment of the [^1^H,^15^N] TROSY‐HSQC experiment, which demonstrates a substantial loss of intensity of the ETR1 signal in the presence of Cu (I) compared with the absence of Cu (I) (Fig. S2). Protein concentrations of apo and Cu(I)‐loaded nanodisc samples were determined by UV absorbance at 280 nm (ε_(280)_ = 208 120 M^−1^ cm^−1^), yielding 97.4 μm and 94.5 μm, respectively. These comparable values confirm that the spectral differences observed in the NMR experiments reflect genuine structural effects of Cu(I) binding rather than differences in incorporation or protein content.

According to mutagenesis [7, 31] and XAS data [30], the essential copper‐binding site is located at residues Asp25, Cys65, His69 in the transmembrane region (TMR). Recent EPR data reveal that the Cu(I) binding site in the N‐terminal TMR of ETR1 is preformed, as copper binding does not cause significant conformational changes [6]. The NMR measurements in this study corroborate the hypothesis that Cu (I) binding in the TMR region stabilizes and rigidifies the ETR1 homodimer, with the rigidification propagating in the C‐terminal part of ETR1, remote to the Cu (I) binding site. This would place ETR1 in line with other metalloproteins, where the binding of metal cofactors has been shown to enhance structural stability and reduce conformational flexibility [32, 33, 34].

## Discussion

In conclusion, NMR analyses in a native‐like membrane environment reveal that the C‐terminal extramembranous domains of the ethylene receptor ETR1 are highly flexible in their apo state. This flexibility can be attributed primarily to the linker regions that connect the GAF, kinase, catalytic, and receiver domains, rather than to disorder within the domains themselves. This interpretation is supported by a series of previous studies demonstrating that each individual domain of the purified full‐length receptor is functional and correctly folded. Circular dichroism (CD) spectroscopy showed that the overall secondary structure content of the full‐length receptor is consistent with sequence‐based structural predictions [28]. The TMD was verified to be properly folded and functional through copper‐binding studies using BCA, EPR, XAS, and EXAFS, as well as through binding assays with a known ethylene analog in the copper‐loaded state [6, 8, 30, 35]. The GAF domain was shown to be functional through extensive peptide‐binding assays with ripening inhibitory peptides such as NOP1 and interaction studies with the receptor's downstream signaling partner EIN2 [36, 37]. Likewise, kinase activity and proper folding of the kinase domain, including its dimerization capability, were demonstrated by phosphorylation assays [27, 28]. Finally, the receiver domain was proven to be functional by interaction studies with the phosphotransfer protein AHP1, as measured by fluorescence polarization (FP) assays [38]. Together, these findings indicate that the observed flexibility in the C‐terminal region arises not from misfolded domains, but from the dynamic nature of the interdomain linkers connecting otherwise stably folded and functional domains.

However, upon Cu(I) binding, the dramatic change in NMR spectra and the reduced number of resonances suggest significant stiffening. We speculate that the kinase catalytic and receiver domains, located C‐terminal to any dimerization interface and connected by an extended loop region, align into a rigid ETR1 homodimer upon Cu (I) addition. In ETR1, aside from the Cu(I)‐binding site in the TMD, the only other physiologically relevant metal‐binding site is found in the nucleotide‐binding domain. Here, the coordination of divalent metal ions such as Mn^2+^, Mg^2+^, or Ca^2+^ is critical for ATP binding and function. Although the role of metal ions beyond this site remains unclear, we cannot rule out their potential involvement in promoting conformational order in otherwise flexible regions of the protein. Supporting this hypothesis, our data show that Cu^+^ addition results in a pronounced rigidification of the full‐length ETR1 receptor. However, whether this effect is specific to Cu^+^ has not yet been systematically tested.

## Conflict of interest

The authors declare no conflict of interest.

## Author contributions

GG and NAL conceived and supervised the study. GG, ML, and NAL designed experiments. ML and NAL performed experiments. GG, ML, and NAL analyzed data. GG, ML, and NAL wrote the manuscript. GG, ML, and NAL made manuscript revisions.

## Supporting information


**Table S1.** Amino acid sequence of ethylene receptor ETR1 with glycine residues marked in red.
**Fig. S1.** Zoom on glycine region of [^1^H, ^15^N] TROSY‐HSQC spectrum of ^15^N ETR1 embedded in DMPC lipid nanodiscs without Cu(I).
**Fig. S2.** Overlay of 1D spectra of the first increments of the [^1^H,^15^N] TROSY‐HSQC experiments on ethylene receptor ETR1, in absence and presence of Cu (I)
**Fig. S3.** Calibration curve of proteins standards on Superose® 6 Increase 10/300 GL column.

## Data Availability

The data that support the findings of this study are available from the corresponding authors georg.groth@hhu.de and nils-alexander.lakomek@hhu.de upon reasonable request.
