# Supplementary material for: Structural dynamics of the plant hormone receptor ETR1 in a native‐like membrane environment
Source: FEBS Lett. 2025 Sep 1;599(22):3381–91. doi: 10.1002/1873-3468.70153 (PMC12643061; doi:10.1002/1873-3468.70153)
Supplement: Supplementary file 1 — Table S1. Amino acid sequence of ethylene receptor ETR1 with glycine residues marked in red. Fig. S1. Zoom on glycine region of [1H, 15N] TROSY‐HSQC spectrum of 15N ETR1 embedded in DMPC lipid nanodiscs without Cu(I). Fig. S2. Overlay of 1D spectra of the first increments of the [1H,15N] TROSY‐HSQC experiments on ethylene receptor ETR1, in absence and presence of Cu (I) Fig. S3. Calibration curve of proteins standards on Superose® 6 Increase 10/300 GL column. [file FEB2-599-3381-s001.docx]

**Supplemental information**

**Structural dynamics of the plant hormone receptor ETR1 in a native-like membrane environment**

Moritz Lemke*^a^*, Nils Alexander Lakomek^b,c,*^, Georg Groth*^a,*^*

*^a^* Institute of Biochemical Plant Physiology, Faculty of Mathematics and Natural Sciences, Heinrich Heine University Düsseldorf, Universitätsstr.1, Düsseldorf 40225, Germany. E-mail: [georg.groth@hhu.de](mailto:georg.groth@hhu.de)

^b^ Institute of Physical Biology (IPB), Faculty of Mathematics and Natural Sciences, Heinrich Heine University Düsseldorf, Universitätsstr.1, Düsseldorf 40225, Germany. E-Mail: [Nils-Alexander.Lakomek@hhu.de](mailto:Nils-Alexander.Lakomek@hhu.de)

^C^ Institute of Information Processing: Structural Biochemistry (IBI-7), Wilhelm-Johnen Str., Forschungszentrum Jülich, Jülich 52428, Germany

**Table S1. Amino acid sequence of ethylene receptor ETR1 with glycine residues marked in red.**


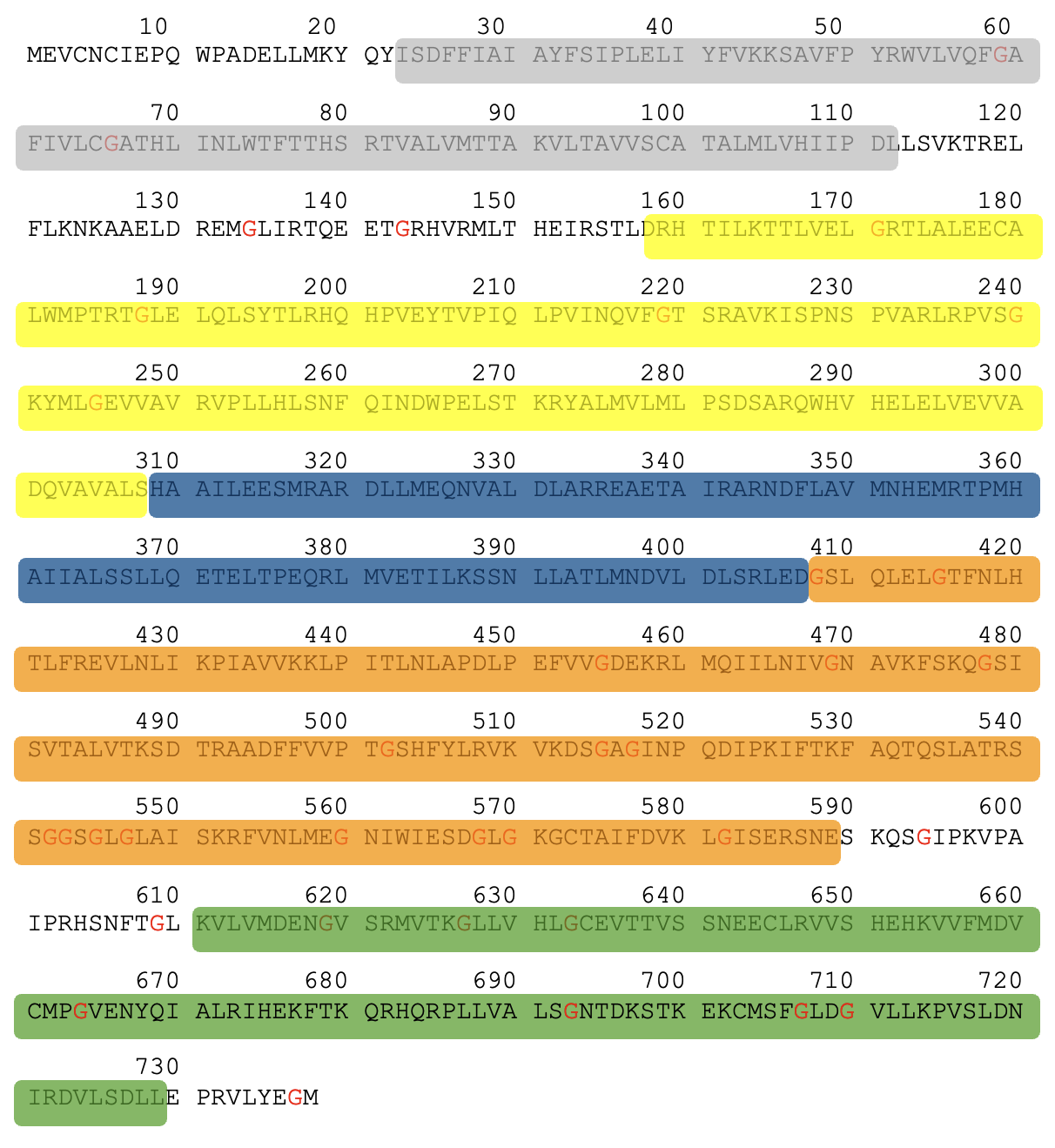


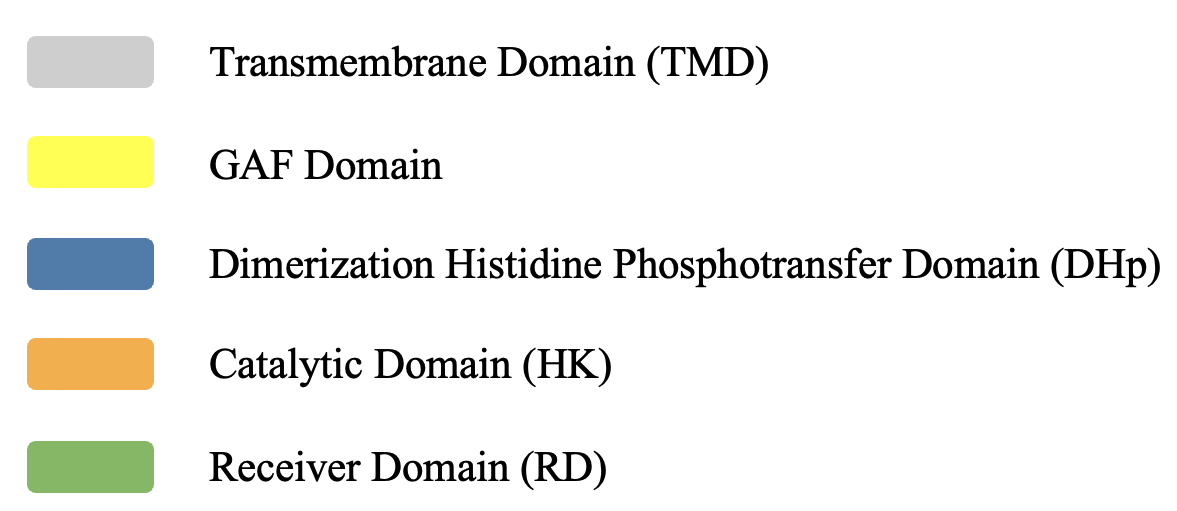


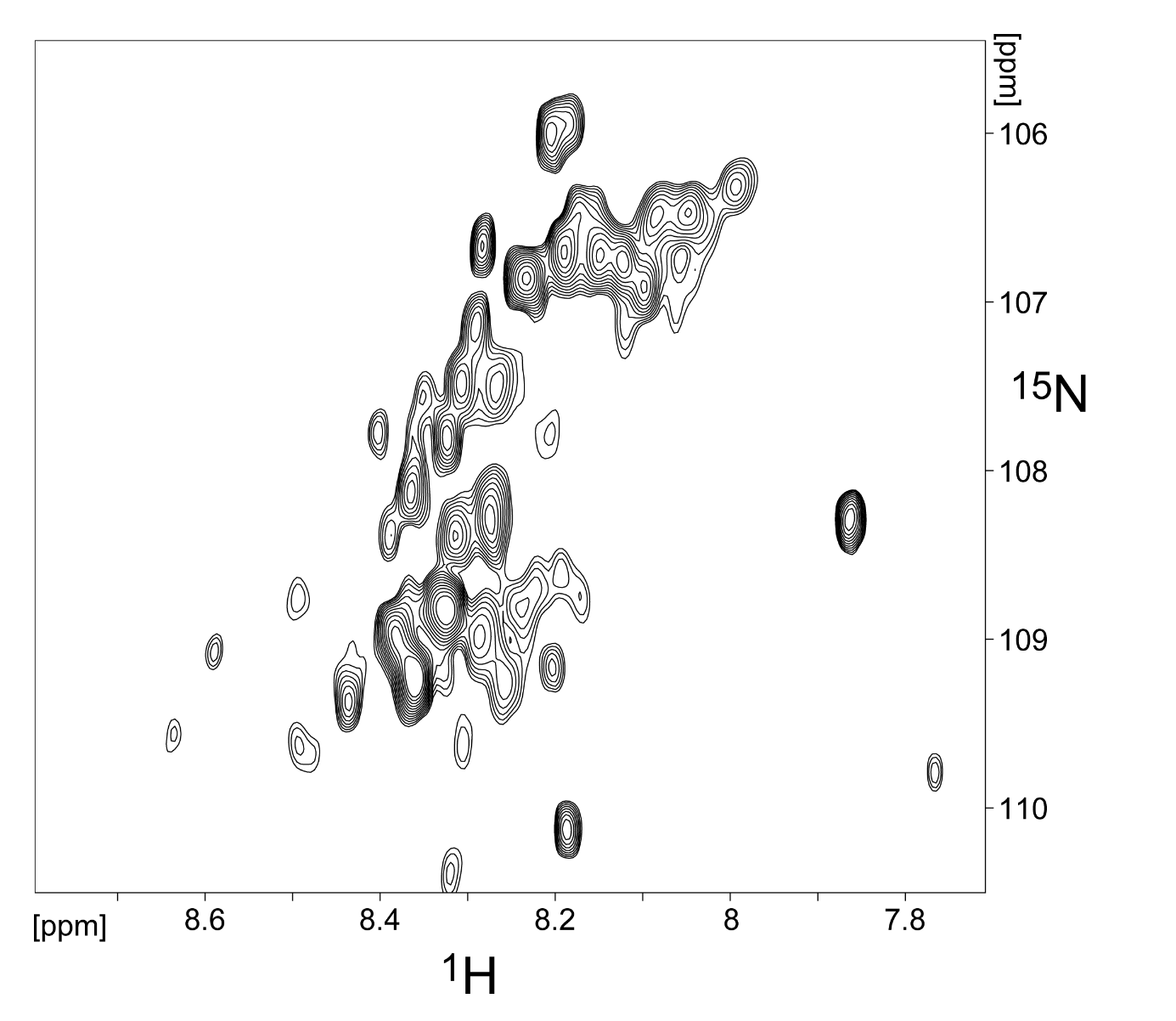
 Figure S1. Zoom on glycine region of [^1^H, ^15^N] TROSY-HSQC spectrum of ^15^N ETR1 (Figure 3A) embedded in DMPC lipid nanodiscs without Cu(I).


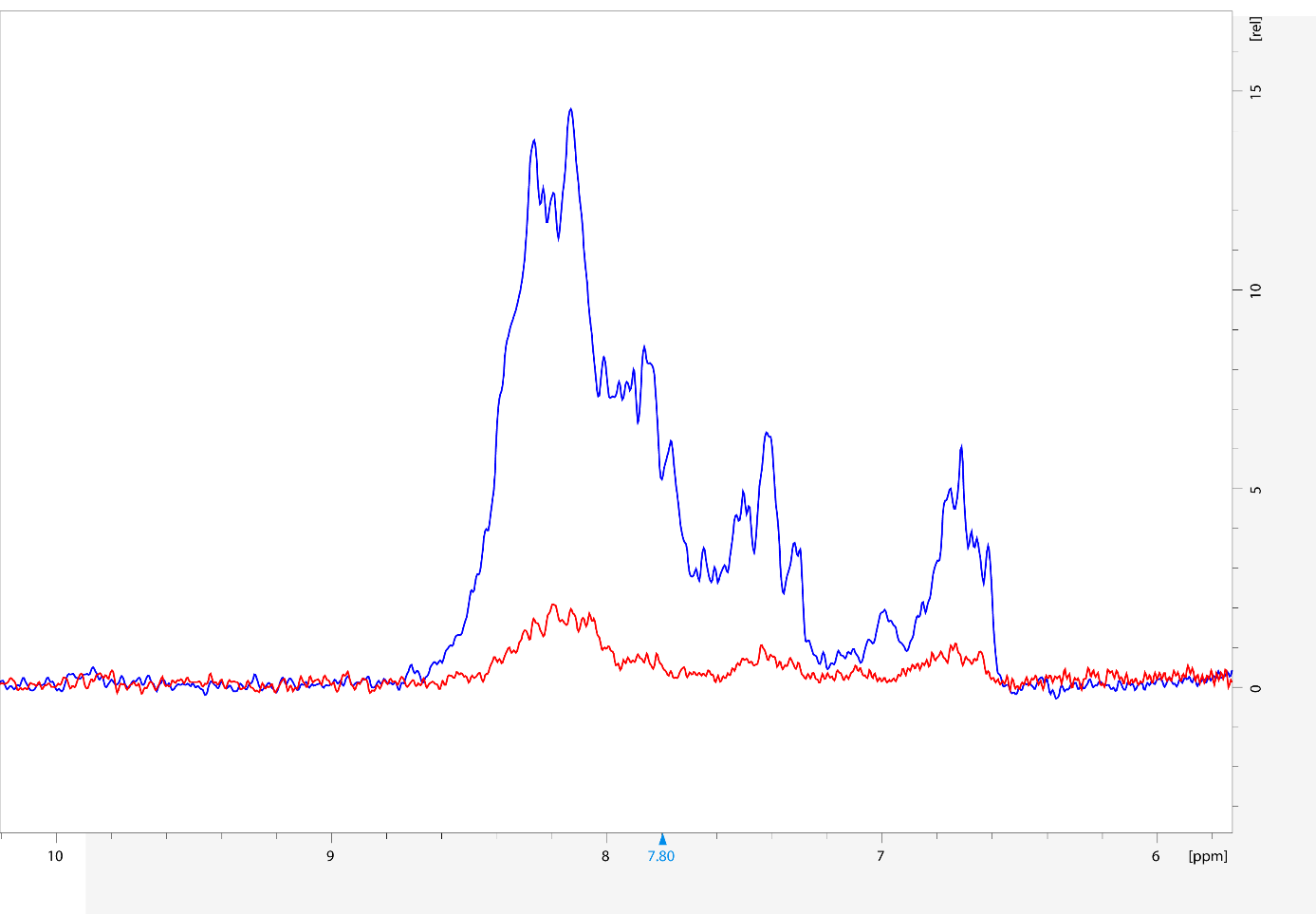
 **Figure S2**. Overlay of 1D spectra of the first increments of the [^1^H,^15^N] TROSY-HSQC experiments on ETR1, in absence of Cu (I) (blue) and presence of Cu (I) (red), shown in Figure 3 A and B.


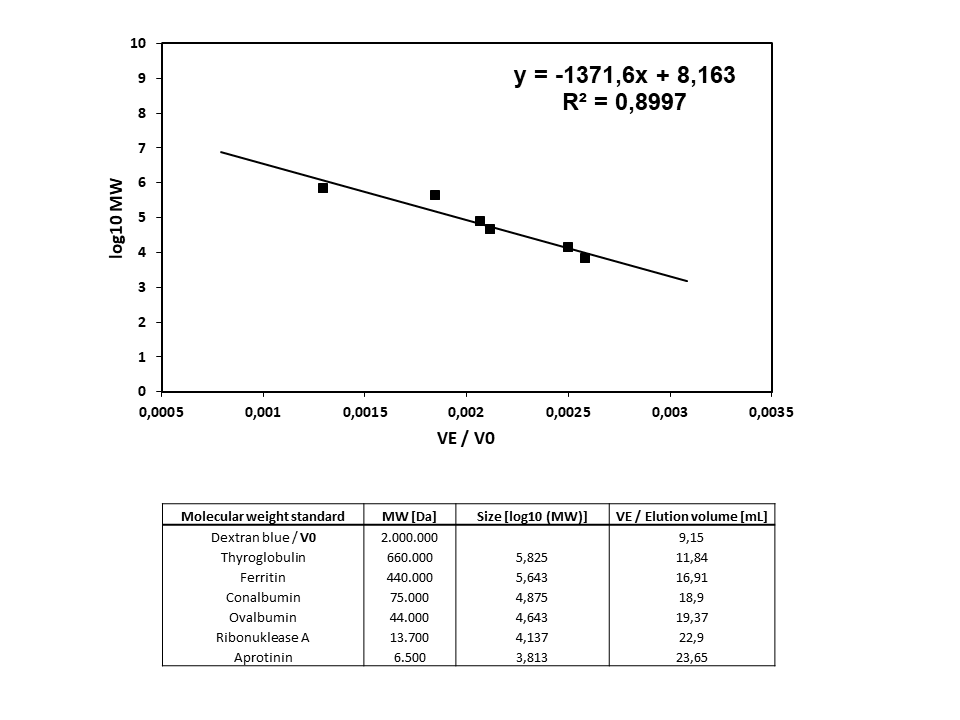
**Figure S3.** Calibration curve (upper) of proteins standards (lower table) using a Superose ® 6 Increase 10/300 GL column at a flowrate of 0.3 mL/min. X Axis: Quotient of elution volume (VE) and void volume (V0). Y-Axis: log10 of molecular weight (Da). The calibration run was performed once under standard conditions and is representative for the determination of the apparent molecular weight of ETR1 nanodiscs shown in Figure 2B.
